# Antibiotic Self-Medication among Non-Medical University Students in Punjab, Pakistan: A Cross-Sectional Survey

**DOI:** 10.3390/ijerph14101152

**Published:** 2017-09-29

**Authors:** Ali Hassan Gillani, Wenjing Ji, Waqar Hussain, Ali Imran, Jie Chang, Caijun Yang, Yu Fang

**Affiliations:** 1 Department of Pharmacy Administration and Clinical Pharmacy, School of Pharmacy, Xi’an Jiaotong University, Xi’an 710061, China; hassangillaniali@yahoo.com (A.H.G.); yfyx_8312@163.com (W.J.); jiechang@mail.xjtu.edu.cn (J.C.); yangcj@mail.xjtu.edu.cn (C.Y.); 2 Center for Drug Safety and Policy Research, Xi’an Jiaotong University, Xi’an 710061, China; 3 The Global Health Institute, Xi’an Jiaotong University, Xi’an 710061, China; 4 Shaanxi Centre for Health Reform and Development Research, Xi’an 710061, China; 5 Faculty of Pharmacy, Bahauddin Zakariya University Multan, Multan 66000, Pakistan; dr.waqarwarraich@gmail.com; 6 Faculty of Pharmacy, University of Lahore, Lahore 54000, Pakistan; aliimran1232002@gmail.com

**Keywords:** self-medication, antibiotics, side effect, resistance

## Abstract

*Background*: Antibiotic resistance is a global threat. Scarce knowledge about safe and appropriate antibiotic use is coupled with frequent self-administration, e.g., in China. This repeated self-medication poses potential risk in terms of antibiotic resistance. Low-resource countries are facing an elevated burden of antibiotic self-medication as compared to developed ones. Thus, this study focused on evaluating the pervasiveness of antibiotic self-medication in 3 universities of Southern Punjab, Pakistan. *Methods*: We conducted a descriptive cross-sectional survey in three government sector universities of Southern Punjab, Pakistan. The study was carried out with self-administered paper-based questionnaires. Data was analyzed using SPSS version 18.0 (IBM, Chicago, IL, USA). *Results*: Seven hundred twenty-seven students out of 750 (response rate 97%) with a mean age ± SD of 23.0 ± 3.4 years agreed to participate in the study. The proportion of females was slightly greater (52%) compared with males (48%), and almost one-third of the respondents (36%) were in their 2nd year of university. Out of the total, 58.3% practiced self-medication in the preceding six months, and 326 (45%) confirmed the use of antibiotics. Metronidazole was the most frequently self-medicated antibiotic (48%). Out of the total, 72% demonstrated awareness regarding the side effects of antibiotics. Diarrhea was the well-known adverse effect (38%). Forty-three percent affirmed having antibiotic resistance knowledge, and 30% knew that the irregular use of antibiotics would lead to increased antibiotic resistance. *Conclusion*: Despite having ample awareness of the adverse antibiotic reactions, self-medication among the university students was high and antibiotic resistance was a fairly unknown term.

## 1. Introduction

In our daily lives, the practice of self-medication (SM) is frequently employed for self-care [1]. The most common definition for SM is the use of non-prescribed drugs by an individual on their own, and it can be further broadened to the treatment of family members, including children and the elderly [2], whereas traditionally it is defined as the utilization of drugs, herbs, and home remedies by one’s own initiative or on the recommendation of others and without consultation with a doctor [3].

WHO affirmed that appropriate and responsible SM can be of assistance to treat and prevent disease more economically and without a doctor’s consultation [4], whereas WHO also reported that inadequate dosing, inappropriate use, incomplete course, increased side effects, and resistance (specifically to antibiotics) and increased tolerance is also associated with unregulated SM [5]. SM is effective if used judiciously, but most of the time it is used erroneously and without proper rationale and guidance. This fact is supported by a previous study in Jordan according to which 67.1% of individuals believed that common cold and cough can be alleviated by antibiotics [6]. Developing countries, compared with developed countries, are encountering an increased self-use of antibiotics; prevalence was reported to be 3% in Europe and 4–75% in the Asia [7,8]. Improper use of antibiotics is a serious hazard in many aspects and disparity in awareness lies between the medical and non-medical community. The awareness is notably high in the medical students compared with non-medical students, and a previous study in Italy demonstrated that only 9.8% among the general population knew about the definition of antibiotics and that 21.3% knew about the appropriate use of antibiotics. In others part of the world, the rate of discontinuation of antibiotics after the patient started feeling better was 49.0% [7,9].

A drastic increase in antimicrobial resistance (AMR), the severity of disease, the duration of the disease, complication risk, mortality rate, and health care cost was observed due to unorthodox use of antibiotics. Among all, AMR is of immense concern [10]. A number of mutated and antibiotic resistant strains are becoming dominant around the world, and developing countries are contributing to strength in resistance [10,11]. In Pakistan, there is an alarming emergence of a “super-bug”, a bacterial strain that is resistant to all available antibiotics [12], and all of this can be credited to unregulated antibiotic use. The strong enforcement of law and public education is of dire need to eradicate the false practice of SM. This was adopted in many developed countries and was effective [8,13]. Antibiotics are prescription-only drugs and their sale should be strictly regulated as “to be sold on a prescription only” basis and should be implemented in reality [14].

Several studies have been performed around the globe to evaluate the prevalence of SM among university students [12,13]. Previous available data, targeting two cities of Pakistan, have revealed that the practice of SM in university students of Karachi increased from 47.6% in 2014 [14] to 80.4% in 2016 [15], while studies from Islamabad showed that SM with antibiotics is almost constant from 2013 (77%) to 2016 (76%) [16,17]. Despite previous activities, the SM rate with antibiotic was constant or increasing, so in order to constitute and implement effective policies and to conduct awareness programs, in other parts of Pakistan, it should be known how common the problem of SM is. Previous studies have focused on particular areas and have not been sufficient for constructing interventional strategies around the country. Students represent an integral part of the community, mainly the educated portion, as they will play different roles in the community and will influence the community. The self-medication practice in students is alarming and ultimately it will spread through the community. These practices should be eradicated from the root level and the intervention or seminars should be conducted to make students aware of the drastic effects of the unorthodox use of antibiotics. This study was conducted to address these problems and to provide a source for appropriate policy measures.

## 2. Materials and Methods

### 2.1. Study Design and Setting

A cross-sectional descriptive study design was adopted to estimate the prevalence of SM with antibiotics among non-medical students in three different universities of Punjab, Pakistan. This study was conducted between July 2016 and September 2016. All of the selected universities were public sector universities with a diverse range of offered programs. In addition to pharmacy, pharmacology, and other medical departments, these universities provide higher education in fields of business administration, agriculture, fine art, economics, and humanities.

### 2.2. Inclusion and Exclusion Criteria

Undergraduate and postgraduate students of any age and gender, familiar with the English language and enrolled in non-medical departments in any of the target universities, were included in the study. Students in paramedical and pharmacy departments and those who were refused to participate in the study were not included.

The use of any kind of medication in the previous6 months of an individual’s own volition without a doctor’s consultation was the definition of SM used in this study. A period of 6 months was selected to minimize bias in recall. This period was considered adequate for one to recall if he had used the antibiotics.

### 2.3. Data Collection Method

To attain the appropriate sample size, the convenient sampling method was adopted. The study objectives were clarified to authorities and prior consent from respective departments was obtained. The data collectors were instructed to approach the students in the target area for interviews. Students taking part in the study gave both written and verbal consent. A self-administered questionnaire was circulated among the study participants, and they were asked to complete it on the spot. The filled questionnaire was then returned to the researchers.

### 2.4. Study Instrument

A 3 section, 8-itempaper-based study instrument, which was validated and used for data collection in previous studies, was adopted [17]. Each section had clear information about the questions to help respondents better understand the nature of each question. Section 1 is comprised of 5 questions regarding the respondent’s demographic, i.e., sex, age, year of university, marital status, and monthly household income. These questions were close-ended, and the result for the demographic variable can be seen in Table 1. Seven items in Section 2 focused on the estimation of the frequency of the SM of antibiotics and the type of antibiotic used in the previous 6 months. A slight description about the definition of SM was made available at the start of this section, and respondents were asked if they engaged in SM in the previous 6 months generally and then specifically with respect to antibiotics. A list of the brand names and their generic name of the most commonly used antibiotics in Pakistan was also included in this section. However, an open-ended extra option of “any other antibiotic used” was also given. The summary of the response to this section is reported in Table 2. Lastly, Section 3 is comprised of 6 questions related to the awareness of side effects due to antibiotic use. Respondents were asked to choose as many adverse effects as they thought might be caused by use of antibiotics. The option of “others” was available if, according to the individual, the adverse effect was not present in the list. This is the only open-ended option in this section. This section was further comprised of two questions aimed to evaluate knowledge regarding the term “antibiotic resistance” and the effect of SM on antibiotic resistance. Tables 3 and 4 show the results of Section 3.

Although this questionnaire was validated and pretested in other parts of Pakistan [17], consistency and validity was further checked in the target population and pilot testing was performed on 90 individuals (30 from each institution). Minute insignificant changes were made afterwards, and the instrument was made available for data collection. Data from pilot testing were not used in the final analysis.

### 2.5. Data Analysis

Data screening and entry was performed by researchers and further cross-checked by colleagues. Data were carefully entered into EpiData 3.1 (Jens M. Lauritsen & Michael Bruus, The EpiData Association, Odense, Denmark) and were analyzed using SPSS (IBM, Chicago, IL, USA). Descriptive statistics was used to calculate the pervasiveness of SM; similarly, frequency of possible reasons for SM, frequency of the SM of different types of antibiotics, knowledge of the most common side effects, frequency of knowledge with respect to antibiotic resistance, and the effect of antibiotic use on resistance was evaluated by generating frequency tables in SPSS.

### 2.6. Ethical Approval

Permission was taken from three universities prior to commencement of the study. Written and verbal consent was sought from each and every individual. Request for the name or any other private info was not made purposefully. This study was approved by the Ethics Committee for Medical Research of Xi’an Jiaotong University Shaanxi, China (ANT-17-21).

## 3. Results

### 3.1. Demographic Characters

A total of 750 students were approached, and among them 727 agreed to take part in the study with are sponse rate of 97%. The mean age ± SD of respondents was 23.0 ± 3.4 years with a minimum age of 18 to a maximum of 40. The proportion of females (52%) was slightly higher than that of males (48%), and the majority of respondents (36%) were in their second year of university. Of the total, 245 (34%) had a monthly household income below 15,000 PKR, and the majority were single (91%). The demographic characteristics were elaborated in Table 1.

### 3.2. Association of Demographic Characters and Prevalence of SM

Out of the total respondents, 326 (45%) stated that they treated themselves with antibiotics without a doctor’s consultation in the previous six months. Gender and year of education showed statistical significance with frequency of antibiotic SM, whereas marital status and monthly household income showed no significance.

### 3.3. Pattern of Antibiotic SM

Metronidazole was the most frequently self-medicated antibiotic amongst the university students, and 156 individuals reported using it. It was followed by ciprofloxacin, amoxicillin and co-trimoxazole. Ampicillin and erythromycin was used by 7% and 6% of the same respondents, respectively, while ampicillin/cloxacillin was amongst the least commonly used antibiotic. The results are shown in Table 2.

Ninety-eight (13%) respondents mistakenly considered other drugs as antibiotics; for example, 78 (11%) participants reported that Panadol was an antibiotic. This was determined by the “other antibiotics” question in Section 2. Here, respondents were asked to report any antibiotic that they used in the previous six months and were not given in the list of antibiotics provided by the researcher.

One hundred seventy-eight (55%) stated that they chose antibiotics for themselves, 34 (10%) followed the instructions of friends, 71 (22%) followed parental instruction, and 46 (14%) followed a pharmacist’s instructions for SM. Out of the total, 571 (78%) knew what antibiotics were, and 522 (72%) of the respondents believed that antibiotics can cause side effects.

**Figure 1:**
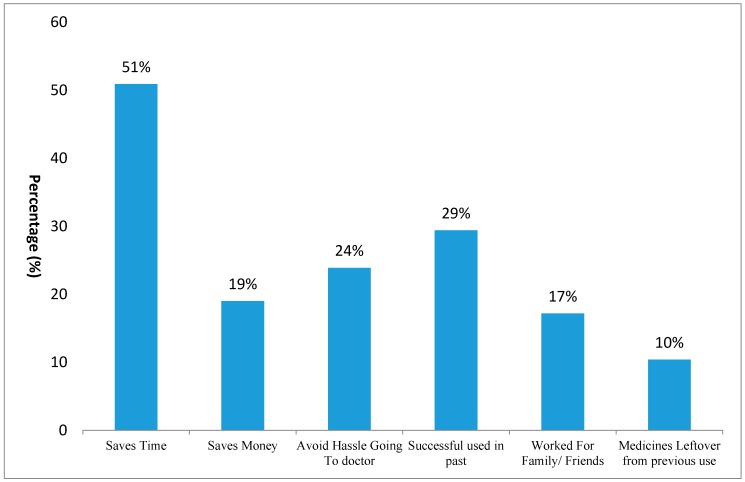
Factors leading to the self-medication (SM) of antibiotics in non-medical students from universities.

Among the 326 who self-medicated themselves with antibiotics, 154 (47%) used them to relieve gastro intestinal problems, 136 (42%) to relieve pain, 118 (36%) to relieve respiratory symptoms, 84 (26%) to treat fever, and 26 (8%) were used to treat urinary problems. Figure 1 contains the reason why respondents self-medicated themselves against any medical condition.

The students were asked about the most common adverse effects that they think can be caused by antibiotics in Section 3. According to the results, the most common side effect reported was by was diarrhea (38%), followed by sleep disturbances (27%), allergic reactions (24%), headache (21%), fever (20%), nausea (17%), tiredness (15%), teeth discoloration (13%), and heart problems (13%). A complete list of awareness of side effects is given in Table 3.

However, 22% of the respondents experienced side effects of antibiotic use. Of 68 students who specified a particular side effect, 18 reported of having gastrointestinal problem after antibiotic use (26%), 16 reported allergic reactions (23%), 13 experienced allergic reactions (19%), 11 experienced sleep problems (16%), and 9 reported headache (13%). Furthermore, the students were then asked if they knew about the term “antibiotic resistance”; 43% (*n* = 311) of the 727 respondents reported that they were aware of this expression and 33% were ignorant of this term. However, it is shown in Table 4 that only 216 out of 727 respondents (30%) knew that the unconventional use of antibiotic is directly related to antibiotic resistance, whereas 44% of respondents admitted that they did not know the relationship between unusual use and antibiotic resistance.

## 4. Discussion

This study provides strong evidence of flaws in policy regarding the sale of antibiotics without a prescription, and this has resulted in an increasing level of SM with antibiotics, as discussed earlier. This study also demonstrated unsatisfactory outcomes in relation to the SM of antibiotics, and it also verified that the burden of SM is high in low- to middle-income countries, as compared to high-income ones [7]. None of the study was conducted in the Punjab province such that it can be used as the baseline for future studies and interventional programs. This study was designed and primarily focused on non-medical university students in Southern Punjab, Pakistan.

The results of this study indicated that 45% of the respondents used antibiotics without consulting doctor in the past six months. The study conducted in the Karachi indicated that 47.0% of students self-medicated themselves with antibiotics; our results were in line with these results [17], whereas a study conducted with university students in the Islamabad showed a prevalence of SM that was much higher than that in our study [18]. A high prevalence of SM with antibiotics was also prevalent around the globe. Research activities indicated that the prevalence of antibiotics was found to be high in Asia [15,19] Africa [20,21], and Latin America [22]. Studies conducted in countries in Middle East regions also showed similar results [9,23].

The factors associated with the self-use of antibiotics were also evaluated in this study. No association was seen between marital status, monthly household income, or the self-use of antibiotics, whereas a statistically significant difference was observed with respect to gender and year of education. The finding in the former studies from Karachi was contrary to our results since none of the demographic parameters had any significant association with the self-use of antibiotics [17], whereas the SM of antibiotics was significantly associated with gender in Sudan and the UAE [24,25].

Several side effects accompany antibiotics when used recklessly and without a doctor’s advice. The three most common reasons for antibiotic SM are that it saves time (166; 51%), that the antibiotic resolved a problem experienced previously (96; 29.4%), and that it bypasses the hassle of seeing a doctor (78; 23.9%). These results are predictable, as it is known that, most of the time, health care costs are exorbitant and that primary, secondary, and tertiary health care facilities are overloaded and botched, unable to fulfill the healthcare needs of individuals. To clarify this situation, further investigative actions regarding patient satisfaction regarding the health care system should be taken. These results coincide with the study in Karachi, where the most common reason for self-medication was that “it saved time” [17]. Another study in Ghana reported that the most common reason was its inexpensiveness, as compared to hospital health care facilities [21].

Most frequent antibiotic used amongst the list given to the students was metronidazole and ciprofloxacin. The results in previous studies in Karachi were in line with our results in that the most frequently used antibiotics was metronidazole [26], while results from another study in Karachi revealed that amoxicilin was the most commonly used antibiotic followed by metronidazole [17]. Gastro complications were the leading symptoms for students self-medicating themselves, and this was followed by pain and respiratory symptoms. Respiratory infections and fever are the most common reasons for antibiotic use in Pakistan [17,19,27], whereas respiratory symptoms are a major reason for self-medication in some other countries [6,15,28].

A small proportion of respondents (24.5%) chose antibiotics for themselves. The target population was a non-medical group, so lack of knowledge about safe and appropriate use rendered them to use antibiotics. A study from Peru showed that the largest portion of the students who self-medicated themselves received recommendations from a pharmacist and that one-fourth chose of their own volition [22]. Further analysis of the knowledge of antibiotics illustrated that almost three-quarters of the respondents were aware that side effects are associated with the erroneous use of antibiotics, and this fact was also apparent in a previous study [17].

More than two-thirds of our respondents, however, were aware of the expression “antibiotic resistance,” and less than two-thirds knew that the unorthodox use of antibiotics will lead to increased antibiotic resistance, while the rest were deprived of the knowledge about the arbitrary use of antibiotics and antibiotic resistance. These results can be explained by the fact that our study population mainly consisted of a non-medical individuals and the term “antibiotic resistance” is generally used in the medical system. The same results were found from the previous study in Karachi [17].

Ultimately, it is the responsibility of government to ensure that any population administers SM responsibly. The government should implement policies to regulate the sale of “prescription-only drugs” upon the presence of prescription. It should be known by the community pharmacist, dispenser, or medical storekeeper that the sale of antibiotics or of any prescription drugs, without prescription, is unethical and is a criminal act. Government, media, and health care authorities should impart their role in educating and motivating users about the safe use of antibiotics. Properly planned educational seminars, lectures, and workshops should be instigated in all universities on anational level. The education should not only focus on the safe use of antibiotics but also potential hazards, the extent of utilization, and accurate dosage.

## 5. Conclusions

The outcome of our study will be supportive in designing programs that will be of assistance in creating awareness of the side effects of antibiotic SM. The pervasiveness of antibiotic SM amongst respondents was high. A large number of study respondents were aware of the possible side effects of the self-use of antibiotics. Nevertheless, the practice of self-utilization of antibiotics was observed.

It is recommended that necessary steps be taken to improve awareness via educational programs, highlighting the risks of unconventional antibiotic use. Physicians should discourage the unnecessary use of antibiotics and prescribe them as a last resort. The health care provider’s role should be to eradicate this practice, especially the community pharmacist who can discourage the purchase of antibiotics without prescription and impart knowledge regarding their safe use. Great negligence on account of the government and health care department was also demonstrated here. The government thus must ensure that the sale of antibiotics is on a prescription basis only.

Our study had few limitations. Firstly, the data is based on university students and thus represent only an educated portion of the general population; however, this sample should be assessed because the educated portion of the community is bound to have the greatest influence on the community. Secondly, we approached students by convenience sampling rather than random sampling. We attempted to restrain the effects by selecting large samples and multiple universities.

## Figures and Tables

**Table 1:** The demographic characters and frequency of self-medication.

Demographic Variable	Students Self-Medicating with Antibiotics (*n* = 326)		Students Not Self-Medicating with Antibiotics (*n* = 401)		*p* Value
	Frequency (*n*)	Percentage (%)	Frequency (*n*)	Percentage (%)	
Gender					
Male	140	43	212	53	0.008
Female	186	57	189	47	
Year in university					
1st	37	11	67	17	<0.001
2nd	123	38	137	34	
3rd	44	13	66	16	
4th	52	16	82	20	
5th	60	18	22	6	
>5th	8	2	25	6	
Marital status					
Single	296	91	367	91	0.290
Married	28	8	34	9	
Divorced	2	1	0	0	
Monthly household income					
<15,000	110	34	135	34	0.415
15,000 to <30,000	80	24	100	25	
30,000–50,000	54	17	82	20	
>50,000	82	25	84	21	

**Table 2:** Frequency of antibiotics that are self-medicated by non-medical university students in Southern Punjab.

Antibiotics	Number of Respondents Self Medicated (%)	Number of Individual Used Once *n* (%)	Number of Individual Used Twice *n* (%)	Number of Individual Used More Than Twice *n* (%)
Metronidazole	156(48)	70 (45)	50 (32)	36 (23)
Ciprofloxacin	108 (33)	68 (63)	20 (18)	20 (19)
Amoxicillin	71 (22)	43 (61)	12 (17)	16 (22)
Co-trimoxazole	62(19)	36 (58)	16 (26)	12 (19)
Ampicillin	24 (7)	14 (58)	8 (34)	2 (8)
Erythromycin	21(6)	15 (71)	0 (0)	6 (29)
Ampicillin/cloxacillin	13 (4)	10 (77)	2 (15)	1 (8)

**Table 3:** Awareness of the side effects caused by antibiotics in the non-medical university students in Southern Punjab.

Symptoms	Frequency (*n*)	Percentage (%)
Diarrhea/abdominal pain	280	38
Sleep problems	196	27
Allergic reaction	177	24
Headache	156	21
Fever	147	20
Nausea/vomiting	123	17
Tiredness	107	15
Heart beat abnormality	95	13
Teeth discoloration	95	13
Yellowness of eyes	85	12.
Muscle pain	80	11
Liver problems	69	9
Numbness	45	6

**Table 4:** Knowledge of the effect of unconventional antibiotic use on antibiotic resistance.

Effect on Antibiotic Resistance	Frequency (*n*)	Percentage (%)
Increase	216	30
Decrease	136	19
Remains the same	52	7
Don’t know	323	44

## References

[1] Kayalvizhi S., Senapathi R. (2010). Evaluation of the perception, attitude and practice of self-medication among business students in 3 select cities, south India. IJEIMS.

[2] Kiyingi K.S., Lauwo J.A. (1992). Drugs in the home: Danger and waste. World Health Forum.

[3] Hernandez-Juyol M., Job-Quesada J.R. (2001). Dentistry and self-medication: A current challenge. Med. Oral Organo Of. Soc. Esp. Med. Oral Acad. Iberoam. Patol. Med. Bucal.

[4] World Health Organization (1998). The Role of the Pharmacist in Self-Care and Self-Medication: Report of the 4th WHO Consultative Group on the Role of the Pharmacist, The Hague, The Netherlands, 26–28 August 1998.

[5] Chughtai S., Khan M.A., ul Haq M.Z., Shahzad A., Hussain F., Nazar F., Chughtai M.A. (2016). Self-Medication amongst the university students of Multan, Pakistan-A questionnaire based survey. Pak. J. Pharm. Res..

[6] Shehadeh M., Suaifan G., Darwish R.M., Wazaify M., Zaru L., Alja’fari S. (2012). Knowledge, attitudes and behavior regarding antibiotics use and misuse among adults in the community of Jordan. A pilot study. Saudi Pharm. J..

[7] Napolitano F., Izzo M.T., Di Giuseppe G., Angelillo I.F. (2013). Public knowledge, attitudes, and experience regarding the use of antibiotics in Italy. PLoS ONE.

[8] Kafle K.K., Gartoulla R.P. (1993). Self-medication and its impact on essential drugs schemes in Nepal: A socio-cultural research project. Action Programme on Essential Drugs.

[9] Belkina T., Warafi A.A., Eltom E.H., Tadjieva N., Kubena A., Vlcek J. (2014). Antibiotic use and knowledge in the community of Yemen, Saudi Arabia, and Uzbekistan. J. Infect. Dev. Ctries..

[10] Llor C., Bjerrum L. (2014). Antimicrobial resistance: Risk associated with antibiotic overuse and initiatives to reduce the problem. Ther. Adv. Drug Saf..

[11] Appelbaum P., Scragg J., Bowen A., Bhamjee A., Hallett A., Cooper R. (1977). Streptococcus pneumoniae resistant to penicillin and chloramphenicol. Lancet.

[12] Bhatti M.W. Emergence of Drug Resistant Bacteria in Pakistan. https://www.thenews.com.pk/print/164414-Emergence-of-drug-resistant-bacteria-in-Pakistan-worries-doctors.

[13] Grigoryan L., Burgerhof J.G., Degener J.E., Deschepper R., Lundborg C.S., Monnet D.L. (2008). Determinants of Self-medication with antibiotics in Europe: The impact of beliefs, country wealth and the healthcare system. J. Antimicrob. Chemother..

[14] WHO Scientific Working Group (1983). Control of antibiotic-resistant bacteria: Memorandum from a WHO Meeting. Bull. World Health Organ..

[15] Sarahroodi S., Arzi A., Sawalha A.F., Ashtarinezhad A. (2010). Antibiotics self-medication among southern Iranian university students. Int. J. Pharmacol..

[16] Ehigiator O., Azodo C.C., Ehikhamenor E.E. (2010). Self-medication with antibiotics among Nigerian Dental Students. Tanzan. Dent. J..

[17] Shah S.J., Ahmad H., Rehan R.B., Najeeb S., Mumtaz M., Jilani M.H., Kadir M.M. (2014). Self-medication with antibiotics among non-medical university students of Karachi: A cross-sectional study. BMC Pharmacol. Toxicol..

[18] Javed M.P. (2013). Self Medication of Antibiotics amongst University Students of Islamabad: Prevalence, Knowledge and Attitudes. Hosp. Pharm..

[19] Pan H., Cui B., Zhang D., Farrar J., Law F., Ba-Thein W. (2012). Prior knowledge, older age, and higher allowance are risk factors for self-medication with antibiotics among university students in southern China. PLoS ONE.

[20] Awad A.I., Eltayeb I.B. (2007). Self-medication practices with antibiotics and antimalarials among Sudanese undergraduate university students. Ann. Pharmacother..

[21] Donkor E.S., Tetteh-quarcoo P.B., Nartey P., Aqyeman P. (2012). Self-medication practices with antibiotics among tertiary level students in accraghana: A cross-sectional study. Int. J. Environ. Res. Public Health.

[22] Núñez M., Tresierra-Ayala M., Gil-Olivares F. (2016). Antibiotic self-medication in university students from Trujillo, Peru. Med. Univ..

[23] Haroun M.F., Al-kayali R.S. (2017). Self Medication among Undergraduate Medical Students in Two Universities in Syria. Int. J. Pharm. Sci. Res..

[24] Abasaeed A., Vlcek J., Abuelkhair M., Kubena A. (2009). Self-medication with antibiotics by the community of Abu Dhabi Emirate, United Arab Emirates. J. Infect. Dev. Ctries..

[25] Awad A., Eltayeb I., Matowe L., Thalib L. (2005). Self-medication with antibiotics and antimalarials in the community of Khartoum State, Sudan. J. Pharm. Pharm. Sci..

[26] Mumtaz Y., Jahangeer S.A., Mujtaba T., Zafar S., Adnan S. (2011). Self-medication among university students of Karachi. JLUMHS.

[27] Hanif A., Ashar S.M., Rabnawaz R., Yasmeen S. (2016). Self-Medication of Antibiotics among the Students of Hamdard University, Pakistan. J. Public Health Dev. Ctries..

[28] Zhu X., Pan H., Yang Z., Cui B., Zhang D., Ba-Thein W. (2016). Self-medication practices with antibiotics among Chinese university students. Public Health.

